# Progress in Surgery

**Published:** 1897-10-09

**Authors:** 


### Progress in Surgery.

### HERNIA.

The introduction of antiseptics and the earlier stage of operation have greatly reduced the mortality in strangulated hernia, the latter being, perhaps, the much more important factor. In the pre-antiseptic era some 30 to 50 per cent. of cases were fatal. Dr. Oscar Hengeller (Zürich) has collected 1,491 cases of the last fifteen years, and finds the mortality to have been 16·5 per cent.[1] Of nearly 300 cases, with a mortality of over 23 per cent., the majority were females, and most occurred between the ages of 50 and 70; labourers being particularly prone to strangulated hernia on the one hand, and child-bearing women on the other hand. Lipomata were present in 9 per cent. of cases. In 12 cases the gangrenous or suspicious-looking bowel was resected with a mortality of over 60 per cent. All the obturator and half the umbilical cases died. Mortality is in direct ratio to the duration of strangulation. Of those operated on within 24 hours only 8 per cent. died. It is, indeed, probable that earlier operation is the cause of the diminishing mortality; this must be obvious from the following facts given by the author, which would be ridiculous in the extreme if antiseptic and aseptic treatment were the sole cause of the diminished mortality: 1881 to 1885, carbolic acid used, 38 per cent. died; 1885 to 1893, bichloride used, 21 per cent. died; since the introduction of aseptic methods only 16·3 per cent. died. Does the author suggest that by using carbolic acid the mortality will be nowadays twice as great as by the aseptic method? Again, how can there be "asepsis" when the fluid in the sac contains bacteria in 30 per cent. of cases? Adolf Brentano has studied this subject, and concludes that, although the bacteria in a hernial sac are few and of low vitality, they occur more frequently than is usually believed, and are present in all cases in which there is much change in the condition of the bowel itself. Micrococci most easily penetrate the bowel wall, and next in order the B. coli commune.

It is known that reducible herniæ and small fatty herniæ of the linea alba give rise to obscure gastric and intestinal symptoms. Schütz, of Vienna, has called recent attention to the relationship between external herniæ and obscure gastro-intestinal disorders.[3] Moreover, the relationship between obesity and hernia is so frequent as to lead to the assumption that excess of fat is often a cause of hernia. Newly-formed fat is liquid at the body temperature, and accordingly exercises mechanical pressure in all directions equally. Pre-hernial lipomata usually precede and cause hernial pouches of the peritoneum; they are, indeed, herniæ of the subperitoneal fat.[4]

**Irreducible Hernia.**—A carefully tabulated collection of 85 cases was made by Mr. Warrington Haward, the causes of irreducibility being usually adherent omentum, or adhesions between omentum and sac, and the examples are only amenable to operation. He concludes that the presence of an irreducible hernia is a condition of serious danger, that the application of a truss does harm by encouraging adhesions, that strangulation of any gut present is very likely to ensue, that herniæ solely of bowel are usually reducible, and that operation is the only method of curing irreducible herniæ.[5]

**Inguinal Hernia.**—Observations have been made by Dr. Packard, Philadelphia, upon the point of origin of oblique inguinal herniæ.[6] He states that the internal abdominal ring exists only in the transversalis fascia, and that there is normally no depression of the peritoneum at this part to be seen from the inside. The sac of the hernia and the sheath of the cord are two distinct tubes, running parallel. Strangulation of the hernia occurs without strangulation of the vessels of the cord, and the valvular aperture in the peritoneum plays an important part by acting as a valve under the intra-abdominal pressure on one side and the distension of the sac on the other. The sac takes its origin in the outer fossa outside the deep epigastric artery, near the true internal abdominal ring, but really above and to its inner side. This view is contested by Dr. Coley.

The statistics of the radical cure of hernia have greatly improved of late, both in regard to diminution of mortality and lessened tendency to relapse. Much of this is due to the stimulus of Bassini, of Padua, who devised his very successful operation, and had only one death and seven relapses in 250 cases. Dr. Coley, New York, reports 360 cases, and speaks in glowing terms of Bassini's operation, especially when kangaroo tendon is used for suturing. The method is equally applicable to femoral hernia.[7] A somewhat gloomy view of the results of radical cure for hernia is taken by Mr. Arbuthnot Lane. He says that herniæ in young children are often dependent upon flatulence and intestinal irritation, the relief of which will cure the hernia. The reduction of the intra-abdominal pressure enables the processus vaginalis to undergo its normal obliteration.[8]

**The Radical Cure of Inguinal Hernia.**—The mortality from this operation for non-strangulated herniæ is very small indeed, a series of 800 cases by four surgeons being without a death. The recorded percentages of relapses vary from 10 per cent. to nil.[9] Mr. McAdam Eccles estimates the relapses at 15 per cent, and gives a table and outline of the cases suited to operation.[10] Heuston points out that the transversalis fascia is the most important structure, keeping the bowel in the abdomen.[11] A method of radical cure without deep sutures has been devised by Duplay and Cazin.[12] It consists in separating the peritoneal sac freely, tying a knot in it as near the abdomen as possible, and then knotting and splicing the remainder of the sac, usually after splitting it into two, or, in some cases, into four parts. When the knotted sac is allowed to fall back, it is found an inch above the internal abdominal ring. Dr. Miller, of Edinburgh, lays stress on the necessity of keeping the patient six weeks in bed, and of never operating on a patient with cough.[13] The comparative advantages and disadvantages of Bassini's and Kocher's operations cannot yet be stated. As Dr. Coley remarks,[14] the fact to be proven before one brought out a new operation for hernia is that Bassini's is more or less a failure. The evidence, however, is all the other way. Again, Bassini's operation has stood the test and scrutiny of several years, whereas no reliable statistics of Kocher's are forthcoming. Vaughan has described an operation in which the conjoined tendon is separated from the rectus, and a new canal for the cord is then formed by sewing the conjoined tendon *in front* of the cord. The old inguinal canal is then stitched up.[15] Coplin Stinson, San Francisco, gives an operation which thoroughly exposes and clears all the inguinal canal, sutures the peritoneum, the internal ring in the transversalis fascia, then sutures the internal oblique and transversalis and their conjoined tendon to Poupart's ligament, and, lastly, stitches the external oblique and the skin.[16] He gives several objections to Bassini's operation and Halsted's modification—chiefly sequelæ of the superficial position of the cord, such as atrophy of the testicle, orchitis, strangulation of the cord, &c.; and he claims at least a maximum theoretical perfection, leaving time to determine its practical value. The radical cure of hernia by implanting a section of sterilised sponge is advocated by Mr. Platt, of Baltimore.[17] The sponge is placed around the cord beneath the internal ring and fixed there, and the canal is closed by sutures. Up to the present there has not been an opportunity of knowing for certain the ultimate fate of the sponge.

An extraordinary case is recorded of a large dependent inguinal hernia, with a spontaneous artificial anus through which there is an eight-inch prolapse of bowel, the woman's anus being literally between her calves.[18]

**Exceptional Inguinal Herniæ.**—Mr. Howard Marsh states a case in which the bowel was so distended with blood that he had to freely incise it before reduction was possible. The patient recovered.[19] Dr. Alfred Smith and Mr. McArdle read notes of a broad ligament myoma which was strangulated in an inguinal canal.[20] Dr. Nelson Irwin described an operation for strangulated inguinal hernia on a child only two months old, who recovered.[21] Mr. Stephen Paget recorded six cases of strangulated herniæ in children, three of whom were only three months old.[22] Mr. W. G. Spencer gave notes of two cases of direct herniæ associated with hernial pouches of bladder.[23] Mr. Chas. Morton described a case in which irreducibility was due to a mesenteric cyst.[24] Dr. Macartney gives details of a variety of operations for 36 cases.[25]

[1] Beiträge zur Klinischen Chirurgie, Band XV., Heft 1. [2] Deut. Zeit f. Chir., XLIII., No. 3, p. 288. [3] Wiener Klinische Wochenschrift, 1896, Vol. 27, p. 595. [4] Bull. de l'Academie de Méd., No. 33, 1896. [5] Lancet, May 29, 1897. [6] Annals of Surgery, July, 1896. [7] Annals of Surgery, March, 1897. [8] Clinical Journal, June 23, 1897. [9] Greiffenhagen (St. Petersburg Med. Woch.), Dec. 28, 1895. [10] Clinical Journal, Nov. 18, 1896. [11] Dublin Journ. Med. Science, Oct., 1896. [12] Sem. Méd., Nov, 1896. [13] Practitioner, May, 1897. [14] Annals of Surgery, May, 1897. [15] Therapeutic Gazette, Nov. 16, 1896. [16] Therapeutic Gazette, Dec. 15, 1896. [17] Johns Hopkins Hospital Bulletin, No. 72. [18] Internat. Journ. Med. Science, May, 1897. [19] Medical Week, May 28, 1897. [20] Brit. Med. Journal, May 29, 1897. [21] N. Y. Med. Record, Nov. 28, 1896. [22] Lancet, Jan. 16, 1897. [23] Brit. Med. Journ., Nov. 21, 1896. [24] Lancet, Dec. 12, 1896. [25] Lancet, Aug. 22, 1896.

